# Effects of Diet and/or Exercise in Enhancing Spinal Cord Sensorimotor Learning

**DOI:** 10.1371/journal.pone.0041288

**Published:** 2012-07-20

**Authors:** M. Selvan Joseph, Zhe Ying, Yumei Zhuang, Hui Zhong, Aiguo Wu, Harsharan S. Bhatia, Rusvelda Cruz, Niranjala J. K. Tillakaratne, Roland R. Roy, V. Reggie Edgerton, Fernando Gomez-Pinilla

**Affiliations:** 1 Department of Integrative Biology and Physiology, University of California Los Angeles, Los Angeles, California, United States of America; 2 Department of Neurobiology, University of California Los Angeles, Los Angeles, California, United States of America; 3 Department of Neurosurgery, University of California Los Angeles, Los Angeles, California, United States of America; 4 UCLA Brain Injury Research Center, University of California Los Angeles, Los Angeles, California, United States of America; 5 Brain Research Institute, University of California Los Angeles, Los Angeles, California, United States of America; University of South Florida, United States of America

## Abstract

Given that the spinal cord is capable of learning sensorimotor tasks and that dietary interventions can influence learning involving supraspinal centers, we asked whether the presence of omega-3 fatty acid docosahexaenoic acid (DHA) and the curry spice curcumin (Cur) by themselves or in combination with voluntary exercise could affect spinal cord learning in adult spinal mice. Using an instrumental learning paradigm to assess spinal learning we observed that mice fed a diet containing DHA/Cur performed better in the spinal learning paradigm than mice fed a diet deficient in DHA/Cur. The enhanced performance was accompanied by increases in the mRNA levels of molecular markers of learning, i.e., BDNF, CREB, CaMKII, and syntaxin 3. Concurrent exposure to exercise was complementary to the dietary treatment effects on spinal learning. The diet containing DHA/Cur resulted in higher levels of DHA and lower levels of omega-6 fatty acid arachidonic acid (AA) in the spinal cord than the diet deficient in DHA/Cur. The level of spinal learning was inversely related to the ratio of AA∶DHA. These results emphasize the capacity of select dietary factors and exercise to foster spinal cord learning. Given the non-invasiveness and safety of the modulation of diet and exercise, these interventions should be considered in light of their potential to enhance relearning of sensorimotor tasks during rehabilitative training paradigms after a spinal cord injury.

## Introduction

Recent evidence indicates that select dietary factors such as docosahexaenoic acid (DHA) can improve learning in the brain by acting on molecular systems involved with synaptic plasticity [1], [2]. DHA belongs to the omega-3 fatty acid family and has important roles in cell communication, synaptic plasticity, and hippocampal learning [3], [4]. DHA has been shown to normalize levels of brain-derived neurotrophic factor (BDNF), reduce oxidative damage, and counteract learning disabilities in animal models of brain trauma [3]. DHA also has been shown to improve locomotor recovery and decrease cellular damage after spinal cord injury. For example, intravenous acute DHA injection combined with dietary supplementation of DHA after a spinal cord compression injury in adult rats decreased the amount of apoptotic cell death and white matter degradation [5]. In addition, the DHA-fed animals showed a significant improvement in their open field locomotor ability at 4 weeks compared to rats receiving either a diet with no DHA or a DHA injection alone. The curry spice curcumin, used in India for many years for several medical purposes, has shown important effects in models of learning and plasticity in animals [6], [7]. For example, curcumin supplementation has been shown to reduce cognitive [8], [9] and locomotor deficits [10], [11] via normalizing levels of BDNF in rodents with brain trauma.

Exercise alone also can improve cognitive learning and locomotor function in intact rats. Volunteer wheel running improved the performance of rats in the Morris water maze that correlated positively with BDNF mRNA levels in the hippocampus [12]. Similarly, long-term locomotor treadmill exercise of intact rats increased BDNF mRNA levels in the lumbar spinal cord compared to sedentary rats [13]. Voluntary wheel running of mice with a spinal cord contusion injury for 3–7 weeks improved their open field locomotor scores [14] and treadmill stepping ability [15] compared to sedentary mice.

Given that BDNF levels are modulated with DHA, curcumin, and exercise, we have focused on molecular systems related to the action of BDNF on synaptic plasticity, such as calcium/calmodulin activated protein kinase II (CaMKII), the gene transcription factor cAMP-response element binding protein (CREB), and syntaxin 3. BDNF is a powerful modulator of neuronal excitability and synaptic transmission [16], [17] and learning and memory in the hippocampus [18]–[20]. BDNF facilitates monosynaptic excitatory post-synaptic potentials (EPSPs) in motoneurons [21]. BDNF mediated activation of CaMKII is a key intermediate in the facilitation of the early phase of long-term potentiation (LTP) leading to a strengthening of synaptic efficacy [22], [23]. CREB is one of the best described stimulus-induced transcription factors involved in gene transcription [24] and memory formation [18]. Syntaxin 3 protein is a pre-synaptic membrane bound protein that participates in vesicular docking that is up-regulated during synaptic plasticity [25]–[27].

With sensorimotor training, the spinal cord can learn to perform both simple and complex motor tasks such as stepping [28]–[32], standing [33], avoiding obstacles [34], and paw withdrawal [35]–[37], even in the absence of supraspinal input [38]. The motor task that is practiced is learned, e.g., if a spinal cat is trained to step, stepping performance is improved [29], whereas if the spinal cat is trained to stand the ability to stand improves [33]. In addition, learning to avoid shock of the paw in an animal with a complete thoracic spinal cord transection has been demonstrated in a variety of species over a period of decades [35], [37], [39]. This spinal learning is mediated via the proprioceptive input to the spinal circuitry, which has the capacity to interpret with considerable detail the patterns of sensory input associated with a given motor task [40]. For example, the spinal cord can interpret sensory changes in load [41] and in treadmill speed [42], [43] and direction [44], [45].

The purpose of the present study was to determine the potential therapeutic role of a combination of DHA and curcumin with and without voluntary exercise in improving spinal learning using the paw withdrawal (PaWL) instrumental learning paradigm in mice [37]. In this paradigm, mice whose spinal cords are completely severed at a mid-thoracic level learn to dorsiflex the paw above a set vertical threshold position to reduce the net shock exposure in response to a mild electric shock applied to the tibialis anterior (TA) muscle. BDNF mRNA levels have been shown to increase with a similar instrumental learning paradigm in spinal rats [36]. Furthermore, delivery of BDNF into the injured spinal cord improves [36], whereas blocking BDNF action hinders the instrumental learning in spinal rats [46], [47]. This PaWL model, therefore, provides the opportunity to evaluate the effects of dietary factors with and without exercise on BDNF-mediated spinal cord learning. The potential benefits of diet and exercise on spinal cord learning have a strong translational potential based on the high efficacy and low invasive profile of these interventions.

## Results

### The effects of DHA/Cur diet and exercise on spinal learning

**Figure 1:**
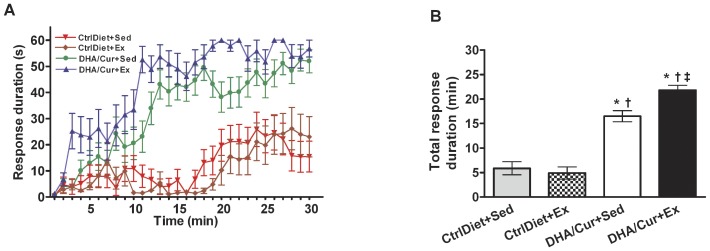
Spinal cord learning evaluated using the paw withdrawal learning paradigm. (A) The groups receiving DHA/Cur with and without Ex had longer mean response durations than both CtrlDiet groups, initially at the 12 min time bin and at almost all time points thereafter during the 30-min PaWL sessions. (B) Mean total response duration in the groups receiving DHA/Cur with and without Ex had longer total response durations than in both CtrlDiet groups. The DHA/Cur+Ex group had a longer mean total response duration than the DHA/Cur+Sed group, reflecting the complementary effect of exercise with the DHA/Cur diet. Values are mean ± SEM (n = 13/group). One-way ANOVA followed by Tukey's post-hoc test at *P*<0.05. ^*^, ^†^, ^‡^: Significantly different from CtrlDiet+Sed, CtrlDiet+Ex, and DHA/Cur+Sed, respectively.

The PaWL paradigm was used to evaluate spinal learning in mice. In general, the mean response duration was longer in the DHA/Cur and DHA/Cur/Ex groups than in both CtrlDiet groups, with these differences being significant at the 12 min time bin and at almost all time points thereafter (Fig. 1A). Total response duration was longer in the DHA/Cur and DHA/Cur/Ex groups than in both CtrlDiet groups (Fig. 1B). In addition, total response time was longer in the DHA/Cur/Ex than DHA/Cur group, reflecting the complementary effect of exercise with the DHA/Cur.

### The effects of DHA/Cur diet and exercise on the levels of molecules associated with learning

**Figure 2:**
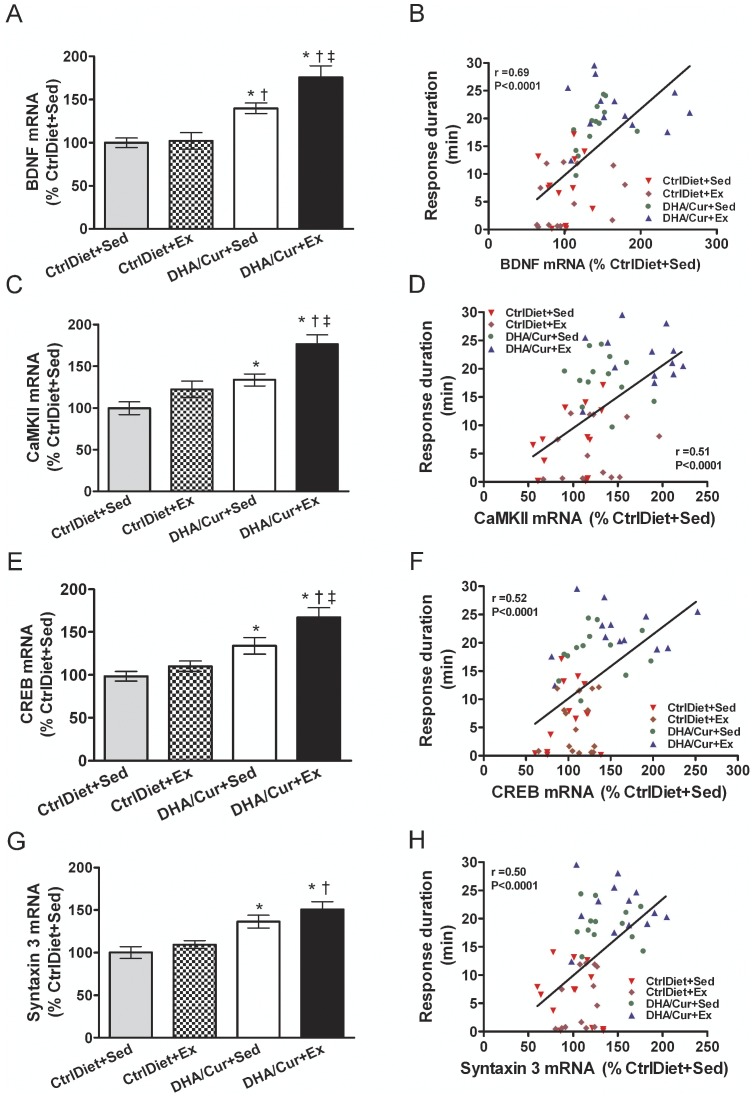
Dietary and exercise effects on the levels of BDNF, CaMKII, CREB, and syntaxin 3 mRNA in the lumbar region of the spinal cord. (A) Mean BDNF mRNA levels were higher in the groups receiving DHA/Cur with and without Ex than in both CtrlDiet groups. Exercise in the DHA/Cur treated group enhanced this response. (C) CaMKII mRNA levels were higher in the DHA/Cur treated groups than in CtrlDiet+Sed group. Exercise in the DHA/Cur treated group enhanced this response. (E) CREB mRNA levels were higher in both groups receiving DHA/Cur than in the CtrlDiet+Sed group. Exercise enhanced the effect in the DHA/Cur group. (G) Syntaxin 3 mRNA levels were higher in both groups receiving DHA/Cur than in the CtrlDiet+Sed group and higher in the DHA/Cur+Ex than in the CtrlDiet+Ex group. There was a significant positive correlation between response duration (spinal learning) and BDNF (B), CaMKII (D), CREB (F), and syntaxin 3 (H) mRNA levels across groups. Values are mean ± SEM (n = 13/group). One-way ANOVA followed by Tukey's post-hoc test and Pearson product correlations between PaWL and mRNA levels. ^*^, ^†^, ^‡^: significantly different from CtrlDiet+Sed, CtrlDiet+Ex, and DHA/Cur+Sed, respectively, at *P*<0.05.

The mRNA levels of BDNF (Fig. 2A), CaMKII (Fig. 2C), CREB (Fig. 2E), and syntaxin 3 (Fig. 2G) were higher in both groups receiving DHA/Cur than in both CtrlDiet groups except that there was no difference in the levels of CaMKII, CREB, or syntaxin 3 mRNA in the CtrlDiet+Ex and DHA/Cur+Sed groups. In addition, the levels of all molecules, except for syntaxin 3, were higher in the DHA/Cur+Ex than DHA/Cur+Sed group, again reflecting a complementary effect of exercise and the DHA/Cur diet. No differences were observed in the CtrlDiet+Sed and CtrlDiet+Ex groups. Response duration across groups was positively and significantly correlated with the mRNA levels of each molecule (Fig. 2B, D, F and H). Thus it appears that there is a complementary effect of adding DHA/Cur to the diet and exercise in promoting spinal learning.

### DHA and AA levels in the spinal cord of each group

**Figure 3:**
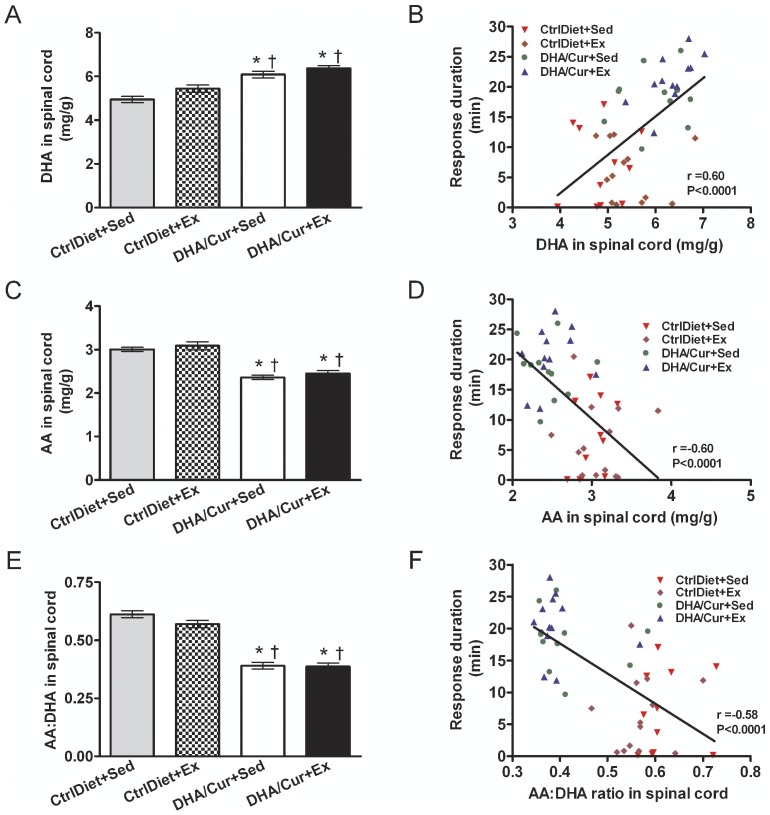
Levels of DHA and AA fatty acids in the spinal cord. The levels of DHA were higher (A) and those of AA lower (C) in the two DHA/Cur groups compared to the two CtrlDiet groups. Consequently, the AA∶DHA ratio was lower (E) in the two groups treated with DHA/Cur compared to the two CtrlDiet groups. There was a significant positive correlation between response duration (spinal learning) and DHA levels in the spinal cord (B) and a significant negative correlation between response duration and AA levels in the spinal cord (D) and the AA∶DHA ratio (F) across groups. Values are mean ± SEM (n = 13/group). One-way ANOVA followed by Tukey's post-hoc test and Pearson product correlations between PaWL and mRNA levels. ^*^, ^†^, ^‡^: significantly different from CtrlDiet+Sed, CtrlDiet+Ex, and DHA/Cur+Sed, respectively, at *P*<0.05.

The spinal cord levels of DHA (Fig. 3A) were higher and those of AA (Fig. 3C) lower in the DHA/Cur+Sed and DHA/Cur+Ex groups than in both CtrlDiet groups. These differences resulted in a lower AA∶DHA ratio in the DHA/Cur+Sed and DHA/Cur+Ex groups than in the two CtrlDiet groups (Fig. 3E). Across groups, there was a positive and significant correlation between mean response time and the levels of DHA (Fig. 3B) and a negative and significant correlation between response duration and both the levels of AA (Fig. 3D) and the AA∶DHA ratio (Fig. 3F) in the spinal cord. Thus the addition of DHA/Cur to the diet results in a change in the fatty acid profile that might promote spinal learning.

### Effects of blocking BDNF function on spinal learning and the levels of molecules associated with learning and plasticity

**Figure 4:**
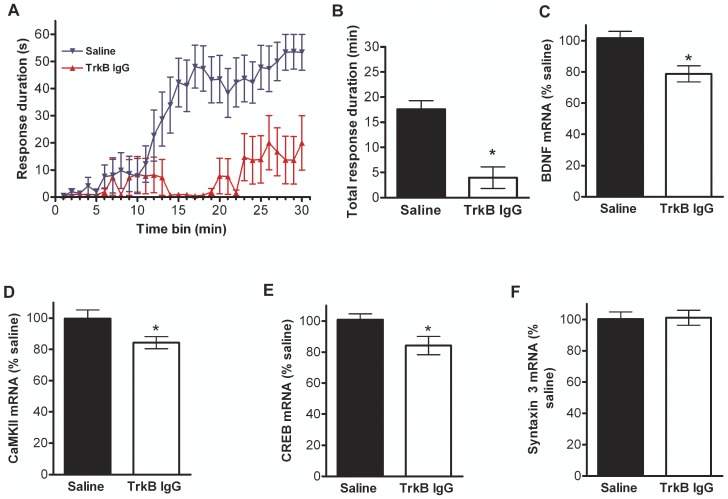
The effect of TrkB IgG on PaWL and the levels of BDNF, CaMKII, CREB, and syntaxin 3 mRNA in the spinal cord. (A) Sequestering of BDNF protein with TrkB IgG significantly decreased spinal learning in mice that were on a DHA/Cur diet. Mean response durations were lower at 13 min and thereafter during the 30-min PaWL sessions in the group receiving TrkB IgG compared to the saline injected group. (B) Mean total response duration was lower in the group injected with TrkB IgG than in the saline injected group. BDNF (C), CaMKII (D), and CREB (E), but not syntaxin 3 (F), mRNA levels were lower in the group receiving TrkB IgG compared to the saline injected group. Values are mean ± SEM (n = 9/group). Mann-Whitney t-test. *: significant difference between Saline vs. TrkB IgG groups, at *P*<0.05.

The lumbar region of the spinal cord of a separate group of spinal mice fed the DHA/Cur+Sed diet was injected with either TrkB IgG or vehicle (saline) 24 hr prior to the PaWL test. The mean response duration in the mice injected with TrkB IgG was significantly lower than in the mice injected with saline beginning at the 13 min time bin and at all time points thereafter (Fig. 4A). Similarly, the mean total response duration (Fig. 4B) and the mRNA levels of BDNF (Fig. 4C), CaMKII (Fig. 4D), and CREB (Fig. 4E), but not syntaxin 3 (Fig. 4F), were lower in the mice injected with TrkB IgG than in the mice injected with saline. Thus an acute decrease in BDNF protein attenuates spinal learning and decreases the levels of plasticity-related markers in the spinal cord.

## Discussion

The principal findings were 1) a diet supplemented with DHA/Cur enhances spinal learning and increases the levels of several learning-related cellular markers, and 2) diet supplementation with DHA/Cur and exercise have complementary effects on spinal learning. Molecular evidence suggests that the spinal learning was mediated through the BDNF pathway as the selective blocking of BDNF signaling with TrkB IgG chimera resulted in decreased learning. In addition, the diet containing DHA/Cur increased DHA and decreased AA levels in the spinal cord: the resulting decrease in the ratio of AA∶DHA was correlated with a greater spinal learning ability.

### Can diet modulate spinal learning?

The dietary effects on spinal learning were accompanied by significant increases in the levels of BDNF, CaMKII, CREB, and syntaxin 3 mRNAs. These results are consistent with previous studies in the brain showing the capacity of DHA [48]–[55] and curcumin [10], [11], [56] to counteract the effects of brain injury on hippocampal-dependent spatial learning performance [11], [55], [57]–[59]. DHA also has shown some beneficial effects when injected in the tail vein 30 min after a spinal cord injury, as indicated by an increase in the survival of neurons and an improvement in locomotor performance after a spinal cord hemisection [60] or compression injury [5].

These results were associated with differential levels of DHA in the plasma membrane. DHA is critical for maintaining membrane fluidity, which is required for proper neuronal function and transmission of information [50], [61]. A reduction in the levels of DHA can lower membrane fluidity leading to dysfunction of transmembrane receptors, potentially affecting the induction of LTP [48] and subsequent learning [49], [62]. Membrane instability and loss of function is suggested by our results showing that the diet with added DHA was associated with an increase in syntaxin 3, a pre-synaptic membrane vesicular transport marker that is regulated by DHA [63]. The levels of both DHA [64] and curcumin [7] can differentially regulate syntaxin 3 in the brain. A deficiency in DHA in the cerebral cortex has been shown to reduce phosphorylation of the BDNF TrkB receptors [52] that, in turn, may reduce the levels of learning-related factors such as CREB [3], [65]. DHA also may influence neurotrophic signaling by activating the PI3-K/Akt pathway that phosphorylates CREB [66]. The increased CREB activation then can influence the expression of other genes including BDNF [4], [18], [67]. In addition, DHA has been implicated in enhancing neurogenesis and plasticity. For example, dietary administration of DHA significantly increased the number of 5-bromo-2(BrdU) ′-deoxyuridine positive newborn neurons in the granule cell layer of the dentate gyrus in adult rats [68], [69]. Exercise also increases hippocampal neurogenesis [70]. Whether the DHA/curcumin diet, exercise, or a combination of both interventions contribute to neurogenesis in the spinal cord was not addressed in the present study. Further investigation using BrdU labeling will be needed to establish whether neurogenesis occurs in the spinal cord as a result of the diet and/or exercise interventions. In the hippocampus, it has been reported that the binding of DHA to GRP40 may provide a fast way to impact plasticity through second messenger pathways such as PLC, PIP3, PKC, and CREB and the c-fos system [71]–[73]. Whether DHA acts through a similar GPR40-mediated mechanism in the spinal cord is unknown and future studies will be designed to address this possibility.

### Role of curcumin in spinal plasticity and learning

We combined curcumin with DHA based on its action in maintaining metabolic homeostasis that can be critical for supporting learning and memory [1], [6], [7]. For example, the beneficial action of curcumin on brain trauma is associated with stabilizing membrane homeostasis and reducing cognitive decay [7], [10]. In addition, curcumin has been shown to reduce cell damage in models of toxicity in culture [9], [74]. The combination of curcumin and DHA in the current study may be particularly effective for fostering plasticity as they act on similar molecular systems. For example, both DHA and curcumin can affect BDNF-related synaptic plasticity [52], [55] and also may influence the metabolic actions of BDNF on glucose and molecules that are crucial for the production of ATP necessary for learning [75], [76].

### Role of exercise and BDNF on spinal learning

The combination of exercise and DHA/Cur promoted a significant enhancement in spinal learning compared to either intervention alone. Exercise positively influences hippocampal-dependent learning via a BDNF-mediated mechanism [12]. In addition, exercise such as wheel running or stepping on a treadmill can improve locomotor recovery after a spinal cord injury [14], [15], an effect mediated at least in part via the BDNF pathway [12], [77], [78]. To confirm that the DHA/Cur diet works through the BDNF pathway, we blocked BDNF function using TrkB IgG in DHA/Cur fed mice. The TrkB IgG injection diminished the spinal learning ability by 3-fold, and reduced the levels of BDNF, CaMKII, and CREB mRNAs in the lumbar segments of the spinal cord. These and other observations [36], [47] support the possibility that the BDNF system is an important intermediate for spinal cord learning. The present results demonstrate that the DHA/Cur diet influenced spinal learning through BDNF-, CaMKII-, and CREB-related pathways. The actions of DHA and curcumin in the brain have been associated with BDNF and TrkB signaling pathways [52], [55], findings consistent with BDNF being an important point of convergence for the beneficial actions of our dietary paradigm on spinal plasticity and learning.

Interestingly, we observed that a diet deficient of DHA/Cur completely suppressed any effect of exercise on spinal learning or molecular markers associated with spinal learning (BDNF, CREB, CaMKII, and syntaxin 3). These results suggest that some DHA must be available in the diet for spinal learning to occur. The results, however, do not indicate how much DHA must be in the diet before an exercise-mediated effect can be detected. We have observed previously that exercise can compensate for the detrimental effects of a diet rich in saturated fat and sugar on the hippocampal levels of BDNF and spatial learning [79], [80].

### Role of the fatty acid profile in the spinal cord on spinal learning

DHA is an essential fatty acid and a structural component of plasma membranes, synaptic vesicles, and membranes of other essential organelles important for brain function and, based on the present results, the spinal cord as well. We observed a decrease in DHA and an increase in the omega-6 fatty acid (AA) in the spinal cord of mice fed a DHA and curcumin deficient diet. The possibility that excessive levels of AA can result in reduction of TrkB signaling is suggested by our results showing that the ratio of AA∶DHA negatively correlated with PaWL performance. In a recent study, deficiency of DHA during brain maturation was associated with reduced TrkB signaling in the brain as well as with increased risk for anxiety-like behavior during adulthood in rats [52]. Overall the evidence seems to indicate that some critical level of DHA and curcumin must play an important role in engaging mechanisms of synaptic plasticity and learning in the spinal cord.

### Conclusions

Our results indicate that a dietary combination of DHA and curcumin facilitates spinal cord learning via a BDNF-related mechanism. The concurrent exposure of exercise during the dietary treatment resulted in an additional improvement in spinal learning. The observation that the spinal learning was proportional to the levels of spinal cord DHA and the DHA-related synaptic marker syntaxin 3, suggest that the action of DHA on the plasma membrane is an important factor for spinal learning as it is in learning tasks involving supraspinal networks [81]. Given that spinal learning can occur in the sensorimotor circuits of the spinal cord and that these circuits can be modulated by dietary modulation of DHA and curcumin, the potential of these interventions for improving recovery after a spinal cord injury should be examined.

## Materials and Methods

The experiments were performed in accordance with the United States National Institutes of Health Guide for the Care and Use of Laboratory Animals. The UCLA Chancellor's Animal Research Committee approved all procedures used in this study.

### Diet and exercise procedures

Adult male C57BL6 mice (Jackson Lab), approximately 10 weeks of age, were housed in standard polyethylene cages in an environmentally controlled room (22–24°C) with a 12 h light/dark cycle. The mice were divided randomly into 4 groups (n = 13/group): (1) Control Diet plus Sedentary (CtrlDiet+Sed); this group served as a control for all comparisons, (2) CtrlDiet plus exercise (CtrlDiet+Ex), (3) DHA plus curcumin plus Sedentary (DHA/Cur+Sed), and (4) DHA/Cur+Ex. After acclimatization for 1 week on standard mice chow, the mice were exposed to either a sedentary or a voluntary exercise condition, with subgroups from each condition exposed to either a Ctrl or DHA/Cur (1.25% DHA; Nordic Naturals, Inc. Watsonville, CA+500 ppm Curcumin: Sigma Aldrich) diet for 21 days. The dose of DHA and curcumin used in this study were the same as used previously in rats [8], [52]. The diets were provided *ad libitum* and administered in powder form.

The two custom diets (CtrlDiet and DHA/Cur) used were based on the composition of the American Institute of Nutrition diet and prepared commercially (Dyets, Bethlehem, PA) as described previously [52] (Table 1). Both diets had the same macronutrients, vitamins, minerals, and basal fats (hydrogenated coconut and safflower oils). The only difference between the Ctrl and DHA/Cur diets was the presence of n-3 fatty acids in the DHA/Cur diet (0.48 and 1.2 g/100 g diet of flaxseed oil and DHA, respectively).

**Table 1:** Dietary Content for CtrlDiet and DHA/Cur diets.

Ingredient	Amount (g/100 g diet)	
	CtrlDiet	DHA/Cur
Alacid 710, acid casein	20	20
Cornstarch	15	15
Sucrose	10	10
Dextrose	19.9	19.9
Maltose-dextrin	15	15
Cellulose	5.0	5.0
Salt-mineral mix	3.5	3.5
Vitamin mix	1.0	1.0
L-cystine	0.3	0.3
Choline bitartrate	0.25	0.25
TBHQ	0.002	0.002

Fat sources		
Hydrogenated coconut oil	8.1	7.45
Safflower oil	1.9	1.77
Flaxseed oil	none	0.48
DHA	none	1.2
Curcumin	none	500 ppm

The running wheel (diameter, 12 cm; width, 5 cm) used for the voluntary exercise rotated freely and was attached to a receiver that monitored the number of revolutions (VitalViewer Data Acquisition System software, Mini Mitter, Sunriver, OR, USA). The mice were allowed to exercise *ad libitum* in individual cages with unlimited access to the running wheel [82]. The amount of exercise wheel running was not significantly different for the CtrlDiet+Ex and DHA/Cur+Ex groups. After 21 days of exercise and/or the respective diets, all mice underwent a spinal cord surgery and PaWL testing.

### Surgical procedures

#### Spinal cord transection

The spinal cord was completely transected at the T7–T8 vertebral level in all mice as described previously [83]. Briefly, under 2% isoflurane anesthesia, a dorsal midline skin incision was made from T6 to T9 and the musculature covering the dorsal vertebral column was retracted to expose the spinal laminae. A partial laminectomy of the T7 and T8 vertebrae was performed to expose the spinal cord. The spinal cord, including the dura, was transected completely using microscissors. The completeness of the lesion was verified by separating the cut ends of the spinal cord with small cotton pellets and by passing a fine glass probe through the lesion site. The skin incision was closed using small surgical staples.

After surgery, the wound sites were treated with triple antibiotic ointment (Bacitracin) and the mice were given lactated Ringer's solution (1.5 ml/30 g body weight, s.c.). Because preventing leg extension during recovery has been shown to facilitate subsequent learning in rats [84], both hindlegs were bound with the knee and ankle joints fully flexed until PaWL testing. The mice recovered in an incubator maintained at 37°C until fully awake and then were returned to their home cages. The mice were allowed to recover for 24 hr before PaWL testing. To minimize bias, the surgeons and testers were blind to the diet and exercise conditions during surgery and PaWL testing.

#### TrkB IgG Injection

A separate cohort of mice were fed the DHA/Cur diet for 21 days and subdivided into two groups (n = 9/group): the spinal cord of one group was injected with physiological saline and the other group with TrkB IgG (R&D Research, rhTrkB/Fc Chimera Cat. # 688). Immediately after the spinal cord transection surgery, the mouse was transferred to a stereotaxic apparatus (David Kopf Instruments, Tujunga, CA). The spinal processes rostral and caudal to the injection site (L2–L3 spinal level) were secured using tissue clamps connected to the stereotaxic apparatus. Injections were made bilaterally at 0.4 mm from the midline and at a 0.32 mm depth [85], [86]. A 10 µl Hamilton syringe fitted with a 100 µm (tip) glass pulled needle was used to inject 0.4 µl of 5 µg/µl of TrkB IgG in saline solution at both sites (total of 0.80 µl) or the same amount of saline as a vehicle control [36].

### PaWL testing

PaWL is a unique paradigm used to demonstrate that the spinal cord can learn a motor task. In this paradigm, a mouse that has undergone a complete mid-thoracic spinal cord transection learns to dorsiflex the paw above a pre-determined threshold when a mild electric shock is applied to the TA muscle. A learned response is determined by the amount of time the paw remains dorsiflexed above the threshold during a 30-min testing period [37].

The PaWL test was conducted 24 h after the spinal cord transection surgery on mice that had received 3 weeks of DHA/Cur+Sed, DHA/Cur+Ex, CtrlDiet+Sed, or CtrlDiet+Ex. The details of the PaWL testing for mice have been described previously [37]. Briefly, during testing the mice were restrained in a closed cloth harness with two slots cut at the end of the harness to allow for both hindlegs to hang freely. Two fine-wire hook electrodes were constructed by removing ∼1 mm of insulation at the end of nylon-coated single strand stainless steel wires (California Fine Wire Co., Grover City, CA). Each wire was passed through a 32-gauge needle: one electrode was inserted intramuscularly into the left TA muscle and the second electrode was inserted subcutaneously at the base of the lateral malleolus on the same side to serve as a ground. The electrodes were attached to a stimulator (S88, Grass Product Group; W. Warwick, RI), through a stimulus isolation unit (SIU5; Grass Product Group) and a constant current isolation unit (CCU1; Grass Product Group). A stimulus duration of 50 msec followed by a 10 msec delay between consecutive pulses was used throughout the PaWL test session as previously described [37].

The stimulation intensity used for each mouse was determined from the current:force curve relationship: we determined the maximum force and then extrapolated to the current required to produce 2/3 of the maximum force. This stimulation intensity then was used for the PaWL testing. We previously have shown this to be the optimal force level needed to elicit instrumental paw withdrawal learning [35], [37]. To determine the optimal force, one end of a silk thread was tied firmly around the distal end of the metatarsals just proximal to the metarsophalangeal joint and the other end was attached to a force transducer (Dual Mode Muscle Lever 300BLR, Aurora Scientific Inc., Aurora, Ontario, Canada). A sequence of stimuli (0 to 1.0 mA at increments of 0.1 mA and with a 30 sec delay between each stimulus) was administered and the force was recorded [37].

To begin the PaWL testing, we positioned the mouse such that its foot was in view of the camera. To identify the resting position of the paw, three rapid priming stimuli were applied at the determined stimulation intensity. The paw position was tracked continuously using a video-based point tracking system (CMUCam2; Carnegie Mellon University). The video information then was converted to two-dimensional axial components for kinematics analysis [37].

A stringent vertical threshold for the paw was set at 1.5 mm above resting position. We initially tested vertical thresholds at 1, 1.5, and 2.0 mm in a pilot study using three mice in each of the CtrlDiet+Sed and DHA/Cur+Sed groups. At a 1 mm threshold both groups learned, indicating that this threshold was not discriminating. At 2 mm neither group learned, indicating that this threshold was too difficult. At the 1.5 mm threshold there was a group difference in the level of learning.

The duration of the PaWL test session was 30 min and the level of learning was assessed by the response duration. The response duration reflects the time that the paw is above the threshold during the PaWL test and incorporates the number of times the foot drops below the threshold resulting in a shock. The response duration is calculated as follows:
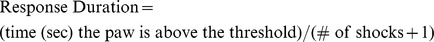



Data collected include the vertical and horizontal paw position, the threshold, and the time when the mice were shocked during all PaWL trials. After completion of the test, the data were post-processed using custom scripts written in MATLAB (The Math Works, Inc., Natick, MA, USA). Data are reported as the response duration for each min binned over the 30-min test (e.g., Fig. 1A) or as the sum of the response duration at each min of the 30-min test (total response duration) (e.g., Fig. 1B). Thirty min after the completion of the PaWL test, the spinal cord was quickly dissected and fresh frozen on dry ice for lipids and mRNA measurements.

### RT-PCR measurements

Total RNA was isolated using the RNA STAT-60 kit (TEL-TEST, Inc., Friendswood, TX, USA) as per the manufacturer's protocol. Total RNA (100 ng) was converted to cDNA using iScript cDNA Synthesis kit (Bio-Rad). The SsoFast EvaGreen Supermix kit was used for qPCR and the cycling conditions were according to the manufacturer's protocol (Bio-Rad). The sequences of the primers were designed using the Integrated DNA Technologies (IDT) online software (“IDT SciTools RealTime PCR”). BDNF: forward (5′- TTACCTTCCTGCATCTGTTGG -3′); reverse (5′- AACATT GTGGCTTTGCTGTCCTGG -3′); syntaxin 3: forward (5′- GCTGGAAGAGATGTTGGA GAG -3′); reverse (5′- TGCTTGGAAATCTGGGAGTC -3′); CREB: forward (5′- ACAGA TTGCCACATTAGCCC -3′); reverse (5′- GAGACTGGATAACTGATGGCTG -3′); CaMKII: forward (5′- CTTTCAGCCAGAGATCACCAG -3′); reverse (5′- ACCAGTAA CCAGATCGAAGATAAG -3′). GAPDH: forward (5′- CTTTGTCAAGCTCATTTCCTGG -3′); reverse (5′- TCTTGCTCAGTGTCCTTGC -3′). The mRNAs for BDNF, syntaxin 3, CaMKII, and CREB were measured using the CFX96 Real-Time PCR Detection System (Bio-Rad). GAPDH gene was used as an endogenous control to standardize the amount of sample loading. The amplification cycle at which the first significant increase of fluorescence occurred was designated as the threshold cycle (CT). The CT value of each sample then was compared with those of the internal standard (GAPDH). The resulting corrected values were used to make comparisons across the different experimental groups. The mean mRNA levels were computed for the four groups. To compare mRNA levels between all experimental groups, we expressed the mRNA levels as a percent of the CtrlDiet+Sed group: the mean mRNA level in each group was divided by the mean of the mRNA level in the CtrlDiet+Sed group and then multiplied by 100 to express the values as a percent (Fig. 2A, C, E, and G). To compare the mRNA levels between the experimental and saline group, the normalization was done similarly and values were expressed as a percent of the saline group (Fig. 4C–F). Error bars for the CtrlDiet+Sed or saline group represent the variation for the individual percent values in that group (Fig. 4C–F).

### Gas Chromatography

Total lipids from entire cervical region of the mouse spinal cord (both sides) were extracted according to the method of Bligh and Dyer [87]. Briefly, the tissues were homogenized with 2 ml lysis solution of chloroform-methanol (2∶1 vol∶vol ) including 0.005% butylated hydroxytoluene. C23:0Me (tricosanoic acid methylester) was added as an internal control. After centrifugation, the liquid was mixed with 0.5 ml 9% NaCl. The chloroform layer containing lipids was collected and dried under nitrogen. Lipids were transmethylated using the BF_3_/methanol reagent (14 wt/v% Boron Trifluoride) at 90° for 1 hr as described previously [52], [55]. Fatty acid composition was analyzed by gas chromatograph (Clarus 500, PerkinElmer, Waltham, MA) equipped with an Elite-WAX column (60 m, 0.32 mm internal diameter, PerkinElmer). The injector and detector temperature was held at 250°C and 300°C, respectively. Hydrogen was used as the carrier gas with a split ratio of 100∶1. Peaks were identified by comparison with fatty acid standards (GLC Reference standard 682, Nu-Chek-Prep, Elysian, MN, and 37-component FAME mix, sigma-Aldrich, Carlsbad).

### Statistics

Data are reported as the mean values ± standard error of the mean (SEM). For comparisons including four groups, a one-way analysis of variance (ANOVA) and Tukey's post-hoc tests were used to determine overall and individual group differences, respectively. Repeated measures ANOVAs were used for the response duration across time bin analyses. Pearson product correlations were used to determine the relationships between response duration (learning) vs. all plasticity markers and the levels of fatty acids in the spinal cord. For comparisons including two groups (BDNF blocking experiments, Fig. 4), unpaired Mann-Whitney t-tests were used. All analyses were performed using GraphPad (GraphPad Software Inc. San Diego, CA). The level of significance was chosen as *P*<0.05 for all comparisons.
